# Role of miRNA-regulated type H vessel formation in osteoporosis

**DOI:** 10.3389/fendo.2024.1394785

**Published:** 2024-05-31

**Authors:** Dailiang Zhang, Yongjing Wang, Zunzhen Zhou, Limei Wang, Chongzhi Liu, Yuan Jiang

**Affiliations:** ^1^ Clinical Medical College and the First Affiliated Hospital of Chengdu Medical College, Chengdu, Sichuan, China; ^2^ Department of Rehabilitation Medicine, The Third Affiliated Hospital of Chengdu Medical College, Chengdu, Sichuan, China

**Keywords:** osteoporosis, miRNAs, osteogenesis-angiogenesis coupling, bone metabolism, type H vessels

## Abstract

Osteoporosis (OP) is a chronic systemic bone metabolism disease characterized by decreased bone mass, microarchitectural deterioration, and fragility fractures. With the demographic change caused by long lifespans and population aging, OP is a growing health problem. The role of miRNA in the pathogenesis of OP has also attracted widespread attention from scholars in recent years. Type H vessels are unique microvessels of the bone and have become a new focus in the pathogenesis of OP because they play an essential role in osteogenesis-angiogenesis coupling. Previous studies found some miRNAs regulate type H vessel formation through the regulatory factors, including platelet-derived growth factor-BB (PDGF-BB), hypoxia-inducible factor 1α (HIF-1α), vascular endothelial growth factor (VEGF), and so on. These findings help us gain a more in-depth understanding of the relationship among miRNAs, type H vessels, and OP to find a new perspective on treating OP. In the present mini-review, we will introduce the role of type H vessels in the pathogenesis of OP and the regulation of miRNAs on type H vessel formation by affecting regulatory factors to provide some valuable insights for future studies of OP treatment.

## Introduction

Osteoporosis (OP) is a chronic systemic bone metabolism disease characterized by decreased bone mass, microarchitectural deterioration, and fragility fractures (1). According to the pathogenesis, OP can be divided into primary, secondary, and idiopathic (2). Primary OP is commonly in post-menopausal women and older people. Secondary OP is caused by certain diseases (e.g., systemic diseases, endocrine diseases, and malignant neoplasms), long-term use of glucocorticoids and other drugs, and lifestyle and habits. In addition, limb disuse, paralysis, and prolonged bed rest can cause bone loss, resulting in OP. Symptoms of OP include shortening of height, hunchback, bone pain, and fragility fractures. However, patients often ignore the characteristic pathological changes of OP before they suffer these symptoms or a bone mineral density (BMD) test is performed. Scholars emphasized that the morbidity of OP increases with age, and OP-related fractures have become a leading cause of mortality in older (3). With the prolongation of lifespan and population aging, OP will continue to increase the fracture risk and medical burden, which is a significant health problem. Scholars have also been committed to exploring interventions to delay the progression of OP and prevent fractures. The in-depth study of the pathogenesis of OP is also crucial for its treatment.

Although the pathogenesis of OP is complex, it is generally accepted that an imbalance between osteoclast-mediated bone resorption and osteoblast-mediated bone formation in the bone remodeling process leads to bone loss and impaired bone microstructure, ultimately resulting in OP. Previous studies have confirmed that estrogen, inflammatory cytokines, growth factors, transcription factors, iron accumulation, and their related signaling pathways can disrupt the balance of bone remodeling by promoting osteoclast differentiation or inhibiting osteoblast proliferation (4–7). In recent years, with the continuous understanding of the role of microRNAs (miRNAs) as epigenetic regulatory factors in controlling gene expression, the role of miRNAs in the pathogenesis of OP has also attracted widespread attention from scholars. Jin and colleagues found that 13 miRNAs were differentially expressed in patients with postmenopausal OP compared with healthy controls, providing evidence that OP is closely related to epigenetics (8). Many studies have confirmed that miRNAs can regulate the process of osteogenesis/osteoclast differentiation by affecting critical regulatory factors and signaling pathways involved in bone metabolism, such as Run-related transcription factor 2 (Runx2), Dickkopf-1(DKK1), RANK/RANKL/OPG pathway, and Wnt signaling pathway (9, 10). These findings provide new thinking about miRNA-based gene therapy in OP treatment.

Bone is a highly vascularized tissue, and its vascular distribution and blood supply play an essential role in maintaining the homeostasis of bone microenvironment and bone remodeling (11). Scholars termed “osteogenesis-angiogenesis coupling” to designate the tight spatiotemporal connection between osteogenesis and angiogenesis. Previous studies have found that bone loss commonly occurs first in the hypoxic area of bone tissue (12). However, hypoxia is also a key driving force for angiogenesis, which can trigger osteogenesis-angiogenesis coupling and enhance bone regeneration, indicating that bone angiogenesis is closely related to bone remodeling (13, 14). An increasing number of studies have confirmed that reductions in the vascular supply of bone tissue are associated with bone loss, indicating that osteogenesis-angiogenesis coupling is involved in the pathogenesis of OP (15, 16). The research on the pathogenesis of OP is no longer limited to bone metabolism. In particular, specific capillaries regulate bone formation by supporting perivascular stem cells or osteoprogenitors. Kusumbe and colleagues discovered a specialized capillary subtype with high expression of platelet endothelial cell adhesion molecule-1 (CD31) and endothelial mucin (Emcn) in the trabecular and cortical bones near the growth plate and on the surface of the periosteum and endometrium of mice, namely type H vessel (17). Type H vessels are densely surrounded by osteoprogenitors that express osteogenesis-specific transcription factors Runx2 and osterix, which maintain osteogenesis by producing specific factors, indicating that Type H vessels play an essential role in osteogenesis-angiogenesis coupling (18, 19). In recent years, type H vessels have become a new focus in OP treatment. Scholars suggested that miRNAs are also involved in osteogenesis-angiogenesis coupling and play a critical role. Some studies have explored the regulation of miRNAs on type H vessel formation, which should help us gain a more in-depth understanding of the relationship among miRNAs, type H vessels, and OP to find a new perspective on treating OP. In the present mini-review, we will introduce the role of type H vessels in the pathogenesis of OP and miRNAs-regulated type H vessel formation by affecting regulatory factors to provide references for the subsequent research on OP treatment.

## Type H vessels and osteoporosis

It is now regarded that the capillaries of the metaphysis and bone marrow cavity can be distinguished into two subtypes, type H vessels and L vessels, by specific cell surface markers Emcn and CD31 (20). The endothelial cells of the former have the characteristic of high expression of CD31 and Emcn, so they are called type H (High) vessels, mainly distributed in the metaphysis and inner periosteum. The endothelial cells of the latter have low expression of Emcn and CD31 and are called type L (Low) vessels, mainly distributed in the diaphysis. Type H vessels form the interconnected columnar network structures that can directly connect to arterial blood vessels, allowing oxygen-rich blood to flow through sinusoidal type L vessels in the diaphysis and finally merge into the venous system (20). The spatial arrangement of type H vessels contributes to them providing proper oxygen and nutrient supply to the bone microenvironment to meet the metabolic demands of osteogenesis (11). Multipotent mesenchymal stem/progenitor cells (MSPCs) are the essential components of the bone marrow microenvironment that can generate osteoblasts, chondrocytes, and adipocytes (21). Coincidentally, more than 82% of Runx2+ and 70% of Osterix+ osteoprogenitors are around type H vessels, while absent around type L vessels. Therefore, although the endothelial cells of type H vessels only account for 1.77% of the endothelial cells of bone tissue, they have crucial promoting effects on bone remodeling (20). In 2017, Langen and colleagues identified another capillary supporting osteogenesis in embryonic and early postnatal bone tissue of C57BL/6J mice, termed type E vessels. However, type E vessels significantly decrease with accompanying skeletal development, possibly due to the conversion processes of type E-to-H vessels (22). In short, type H vessels serve as a crucial link between angiogenesis and osteogenesis.

In addition to identifying the type H vessels, Kusumbe and colleagues found that the number of type H endothelial cells and osteoblasts decreases in experimental mice with aging, indicating that age-related OP may be related to the reduction of type H endothelial cells (17). Other scholars also found that type H endothelial cells decreased in other rodent models of different types of OP, such as postmenopausal OP (23), glucocorticoid-induced OP (24), disuse OP (25), and diabetic OP (26). In human subjects, Wang and colleagues first demonstrated the type H vessel is present in the proximal femur near the greater trochanter and coupled with osteoprogenitors. They also found that reduced type H vessels are associated with age and suggested that the abundance of type H vessels is an early sign of bone loss (27). Zhu and colleagues found that the percentage of type H endothelial cells was positively associated with the BMD of the femur neck and total hip in women. The percentage of type H endothelial cells in postmenopausal women suffering bone loss was significantly lower than in premenopausal women. They suggested that type H endothelial cells can be the markers of bone loss and potential therapeutic targets for OP (28). Promoting type H vessel formation to increase bone mass may be a feasible treatment strategy for OP treatment.

## miRNAs and type H vessel formation

**Figure 1:**
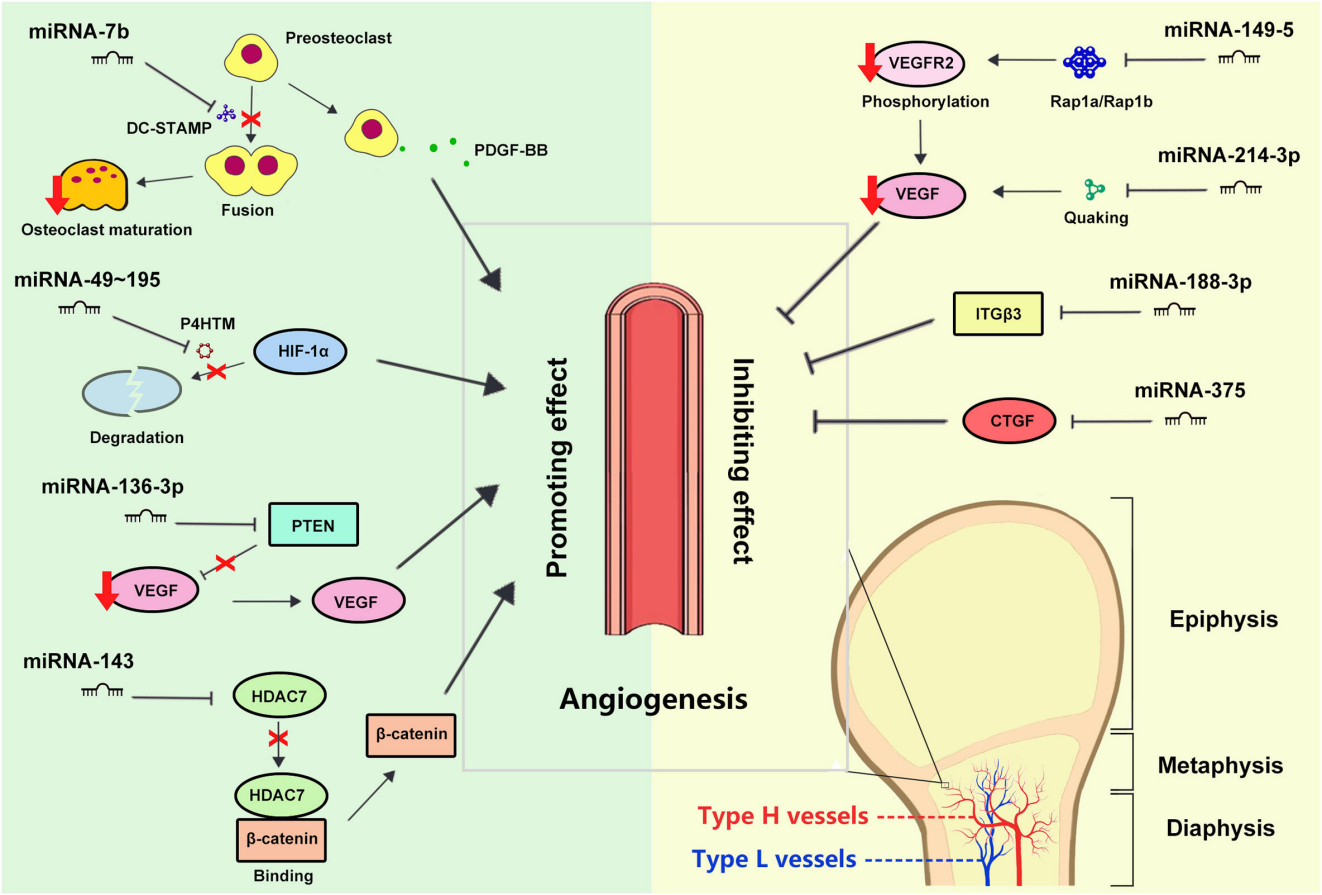
Schematic representation of the regulation of type H vessel formation by miRNAs.

Existing experimental evidence has already proved several factors can regulate type H vessel formation, such as platelet-derived growth factor-BB (PDGF-BB), hypoxia-inducible factor 1α (HIF-1α), vascular endothelial growth factor (VEGF), and so on (20). Some of these regulatory factors can be affected by specific miRNAs, indicating that miRNAs play a role in regulating type H vessel formation, which also explains the involvement of miRNAs in osteogenesis-angiogenesis coupling (**Figure 1**).

### PDGF-BB

PDGF-BB is the most active and functional subtype of the PDGF family, which is involved in chemotaxis and mitosis as a chemotactic and mitogenic factor. PDGF-BB promotes the migration, proliferation, and differentiation of various mesenchymal cells involved in angiogenesis and osteogenesis (29, 30). Xie and colleagues found that serum and bone marrow levels of preosteoclasts-derived PDGF-BB and numbers of type H vessels decreased in ovariectomized (OVX) mice. In contrast, the local injection of exogenous PDGF-BB in the femur or systemic delivery of inhibition of cathepsin K (L-235) increased PDGF-BB concentrations and induced type H vessel formation depending on Akt-dependent activation of FAK signaling. They suggested that the secretion of PDGF-BB by preosteoclasts is a critical step of H vessel formation during bone modeling and remodeling (31). However, an increase in mature osteoclasts in OVX mice also implies a decrease in preosteoclasts and their secretion of PDGF-BB. Dou and colleagues used miRNA-7b to reduce the expression of the critical fusogenic molecule directly, dendritic cell-specific transmembrane protein (DC-STAMP), for inhibiting mature osteoclasts formed by the fusion of preosteoclasts during osteoclast maturation. They found that the overexpression of miRNA-7b did not affect the preosteoclast formation but contributed to maintaining preosteoclasts and keeping their secretion of PDGF-BB to increase type H vessel formation in OVX mice (32).

### HIF-1α

HIF-1α is a subunit of HIFɑ triggered by hypoxic states and serves as a critical regulatory factor in the adaptation of cells to hypoxia. Under normoxic conditions, HIF-1 α may undergo rapid degradation by proteases in the cytoplasm after being hydroxylated by prolyl hydroxylase (PHDs) under normoxic conditions. However, HIF-1α can exist steadily by PHD inactivation and regulate the expression of VEGF under hypoxic conditions, which induces endothelial cell proliferation and promotes angiogenesis (33). Kusumbe and colleagues found high HIF-1a expression in the type H endothelial cells but not in the type L endothelial cells in the bones of the 2-week-old mouse. They used a PHD inhibitor (deferoxamine mesylate, DFM) to reverse the lower HIF-1α expression in the type H endothelial cells of aging mice, leading to angiogenesis of type H vessels and enhanced type H vessel abundance (17). Yang and colleagues found that the miRNA-49~195 clusters also had high expression in the type H endothelial cells in C57BL/6 mice and gradually decreased during aging. Compared to wild-type (WT) mice, miRNA-497~195 cluster knockout mice showed fewer type H vessels, but miRNA-497~195 cluster transgenic mice showed an increased number of type H vessels by overexpressing miRNA-497~195 in endothelial cells that contributed to type H vessel formation, indicating that the miRNA-497~195 cluster was involved in regulating type H vessel formation. They also found that overexpressing the miRNA-497~195 cluster increased HIF-1α protein levels but not the mRNA levels. They suggested that overexpressing the miRNA-497~195 cluster inhibited the expression of P4HTM, one member of the PHD family, which contributed to maintaining HIF-1α protein stability in endothelial cells to promote type H vessel formation (34).

### VEGF

VEGFA is a subtype of the VEGF family, commonly known as VEGF, and is a critical mediator of angiogenesis. VEGFA binds to VEGFR2 receptors to promote endothelial cell proliferation, migration, and angiogenesis (35). In addition to vascular endothelial cells, other cells in the bone microenvironment can secrete VEGFA, such as osteoblasts, osteoclasts, chondrocytes, and even immune cells (36). Some cytokines and transcription factors are involved in the transcription and secretion of VEGFA, such as HIF-1α, PDGF-BB, Noggin, and Runx2 (20). Previous studies showed that VEGFA is also a critical downstream effector of PDGF-BB, which can stimulate type H vessel formation in bone tissue besides HIF-1α (37). Some scholars suggested that miRNAs can inhibit the formation of type H vessels by down-regulated VEGF. Wang and colleagues reported that high expression of miRNA-214–3p negatively regulates the formation of type H vessels. They found that miRNA-214–3p increased in the VOX mice by miRNA screening and sequencing, but high levels of miRNA-214–3p decreased the expression of VEGF. Contrarily, the inactivation of miRNA-214–3p positively affects the formation of type H vessels by reversing the low levels of VEGF (38). In addition, She and colleagues suggested that miRNA-149–5p decreased the expression of VEGF via the Rap1a/Rap1b/VEGFR2 signaling axis, which can impair the formation of type H vessels. Targeted inhibition of miR-149–5p may be an effective way to promote type H vessel formation (39). CTGF.

Connective tissue growth factor (CTGF, also called CCN2) is a cysteine-rich matricellular protein expressed in the vascular wall, which is involved in chondrogenesis and angiogenesis during skeletal development (40, 41). He and colleagues reported that when they explored the effect and mechanism of prenatal caffeine exposure on long-bone development in female fetal rats, they found that CTGF expression decreased in the growth plate induced the inhibition of angiogenesis of H-type endothelial cells in female fetal rats (42). They suggested that chondrocyte-induced up-regulation of the miR375 expression is the molecular mechanism of the reduction in CTGF expression. The application of miRNA-375 inhibitor can reverse the inhibition of CTGF expression induced by caffeine and the adverse H-type blood vessel formation, indicating that miRNA-375/CTGF can mediate H-type blood vessel formation.

### Integrins

Integrins, as transmembrane proteins, are the leading receptors of the extracellular matrix (ECM) and can mediate the attachment of cells to the ECM (43, 44). For endothelial cells, adhesion to ECM via cell-surface integrins is necessary for endothelial cell proliferation and angiogenesis (45). Previous studies showed that integrins, such as integrin β1 and integrin β3, also exist in type H endothelial cells and play a crucial role in angiogenesis (46). He and colleagues found specific overexpression of miRNA-188–3p in the senescent endothelial cells of type H vessels, which inhibits the type H vessel formation, especially in aged mice. They predicted the potential targets of miRNA-188–3p using online bioinformatics tools and found that integrin β3 (ITGβ3) is the direct target. They suggested that miRNA-188–3p directly targets integrin β3 in endothelial cells, inhibiting type H vessel formation and contributing to age-related type H vessel decline. By contrast, knocking out miRNA-188 can alleviate the type H vessel decline in aged mice (46).

### Histone deacetylase 7

Histone deacetylase 7 (HDAC7) is a subtype of class II HDACs and is a zinc-dependent enzyme (47). HDAC7 can affect the differentiation and maturation of osteoblasts by associating with Runx2 and suppressing the activity of Runx2 in the osteocyte (48). HDAC7 is also present in vascular endothelial cells and can inhibit endothelial cell growth while maintaining a low proliferation stage by directly binding to β-catenin and suppressing β-catenin activity. Notably, HDAC7 is also critical for VEGF-induced endothelial proliferation as a downstream respondent of VEGF signaling (49). VEGF can induce HDAC7 degradation and decrease the combination of HDAC7 and β-catenin, resulting in endothelial cell growth (50). HDAC inhibitors (HDACi) such as valproic acid (VPA) and suberoylanilide hydroxamic acid (SAHA) can also increase the expression of β-catenin, contributing to capillary-like sprouting of endothelial cells (51). Some scholars found that HDAC7 silencing increases the secretion of PDGF-BB protein in endothelial cells (52). These findings indicated that HDAC7 is involved in osteogenesis-angiogenesis coupling as a negative regulator. Wang and colleagues found that the expression levels of miRNA-143 in serum from aged patients with fractures were lower than those of younger patients, indicating that the serum miRNA-143 level was negatively correlated with age and may be involved in bone loss in age-related OP (53). They also observed the expression of miRNA-143 in the type H endothelial cells was higher than that in type L endothelial cells, and there was a significant decrease in type H endothelial cells in miRNA-143-knockout mice. The results of mRNA-sequencing analysis indicated HDAC7 was the potential target of miRNA-143. Inhibition of miRNA-143 enhanced HDAC7 protein expression, but HDAC7 knockdown can rescue the decreased type H endothelial cells in miRNA-143-knockout mice, indicating that miR-143 promoted type H vessel formation by targeting HDAC7.

### PTEN

Phosphatase and tensin homology deleted from chromosome 10 (PTEN) is a tumor suppressor gene located on chromosome 10q23 (54). Its encoded PTEN protein is a lipid phosphatase that regulates cell growth, differentiation, migration, and apoptosis (55). PTEN can negatively regulate cancer cell-induced angiogenesis and inhibit VEGF-mediated endothelial cell proliferation in the normal vascular, indicating that PTEN also serves as a critical mediator in angiogenesis (56, 57). Chen and colleagues found that high expression of PTEN in the animal model of alcohol-induced osteopenia may play an indispensable role in the negative effect on angiogenesis of bone tissue by long-term and excessive alcohol consumption, especially type H vessels (58). Interestingly, they identified that miRNA-136–3p was remarkedly downregulated in the animal model of alcohol-induced osteopenia, while miRNA-136–3p can directly modify PTEN expression. Then, they abolished PTEN protein and restored the decreased number of type-H vessels in the ethanol-treated animals after the supplement of miRNA-136–3p, suggesting that miRNA-136–3p plays a protective role in maintaining type-H vessels via miRNA-136–3p/PTEN axis.

## Exosomal miRNAs and osteoporosis

**Figure 2:**
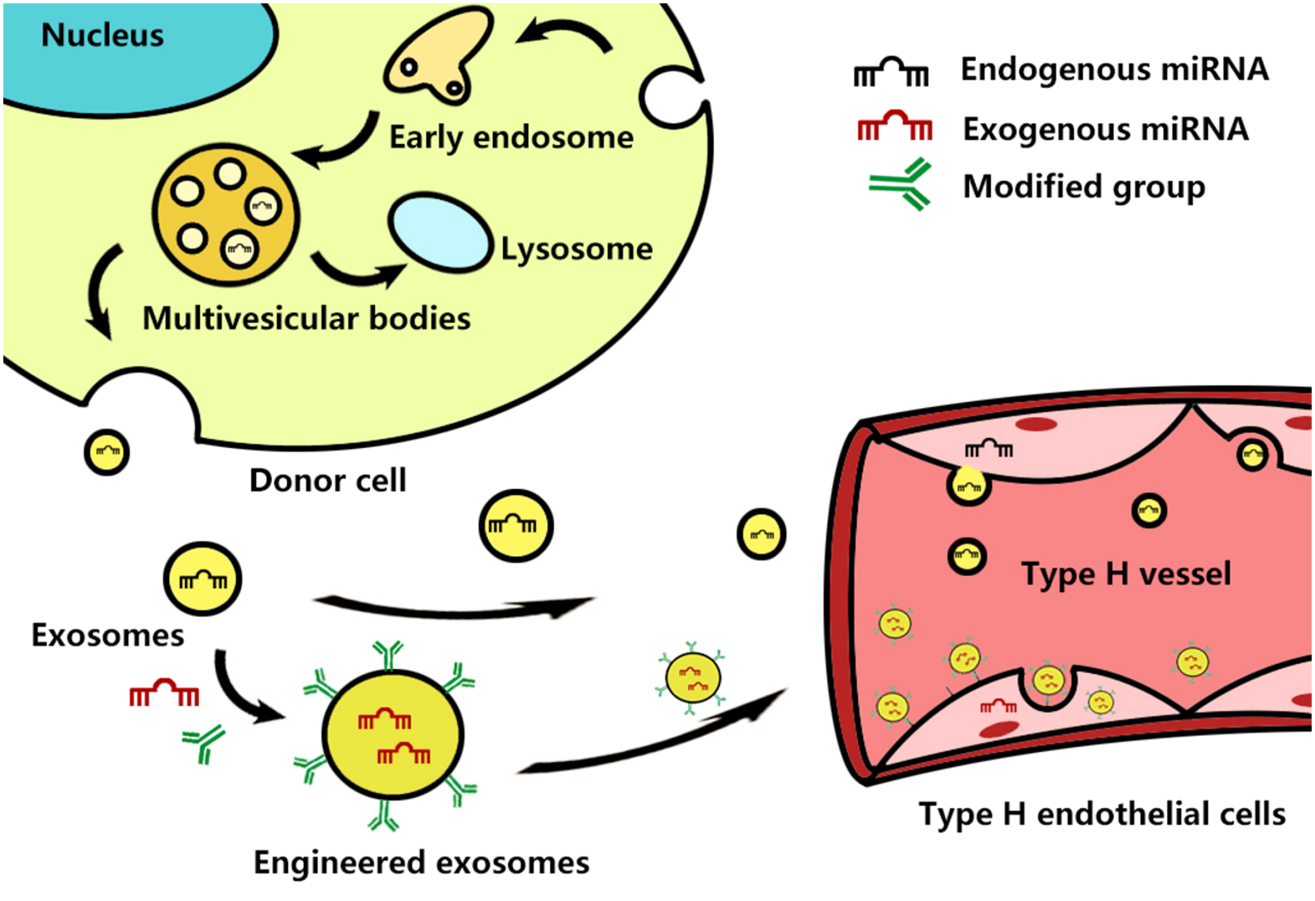
Schematic representation of exosome biogenesis and how they and engineered exosomes interact with the type H endothelial cells. Exosome biogenesis starts from the inward budding of the cellular plasma membrane to form early endosomes, which then mature into late endosomes. Late endosomes bud into intraluminal vesicles (ILVs) and multivesicular bodies (MVBs) and encapsulate endogenous miRNAs and other bioactive molecules. After fusion with the cell membrane, MVBs are released into the intercellular space as exosomes, while the other MVBs fusion with lysosomes to degrade. The exosomes and exogenous miRNA-containing engineered exosomes enter the blood circulation, attach to the cell surface of type H endothelial cells, and then are uptake by the host cell.

It is noteworthy that some miRNAs come from the above-introduced come from exosomes. For example, miRNA-214–3p comes from bone marrow-derived mesenchymal stem cells (BMMSCs), and miRNA-149–5p comes from the extracellular vesicles derived from zoledronic acid-treated bone marrow-derived macrophages. Recently, Wang and Xu found the TGF-β1-stimulated BMSC-derived exosomes carrying highly expressed miR-135b inhibit the type H vessel formation by down-regulating PDGF-BB expression (59). This finding has drawn our attention to exosomes, a subtype of extracellular vesicles (EVs) with 40–200 nm nano-size, which are secreted by almost all cells, including various types of cells for constructing bone microenvironment (60, 61). Exosomes contain many constituents of the donor cells, including protein, nucleic acids, lipids, amino acids, and metabolites (62). MiRNAs are an essential ingredient of exosomal nucleic acids and can be taken up by neighboring or distant cells to modulate recipient cells, contributing to achieving exosome-based intercellular communication (63) (**Figure 2**). Previous research has confirmed that exosomal miRNAs are also closely associated with the development of OP. Shao and colleagues found that a total of 191 exosomal miRNA aberrant expression profiles existed in the group of menopausal women with OP in contrast to menopausal women without OP, including 72 upregulated miRNAs and 121 downregulated miRNAs. These miRNAs are involved in many signaling pathways, such as the Wnt, mitogen-activated protein kinase (MAPK), and Hippo pathways, indicating that serum exosomal miRNAs were associated with postmenopausal OP (64). Of course, exosomes derived from different cells or cells with various functional statuses might induce diverse effects on the development of OP. MSCs-derived exosomal miRNA-196a stimulate osteoblast activity to promote bone formation, while osteoclast-derived exosomal miR-214–3p inhibits osteoblastic bone formation by inhibiting osteoblast activity (65, 66). Song and colleagues found that vascular endothelial cell-secreted exosomes specifically target bone tissue by pregnancy zone protein (PZP) and inhibit osteoclast formation by delivering miR-155 to macrophages to blocked Spi1, microphthalmia-associated transcription factor (Mitf), and suppressor of cytokine signaling 1 (Socs1) mRNA and protein expression (67). Still, many scholars believe exosomes are a promising new strategy for treating OP. Exosomes can also affect vascular endothelial cells with their miRNAs to promote angiogenesis in bone tissue, which may be another potential advantage of the exosome-based treatment strategy for OP (68). In addition, exosomes can be easily surface-modified and encapsulate therapeutic agents (e.g., drugs, siRNA, miRNA, and proteins) using various engineering methods (69). Engineered exosomes can deliver special exogenous miRNA to the bone microenvironment, which can take priority in selection (70). Hence, We consider that engineered exosomal miRNAs can contribute to achieving targeted type H endothelial cells to regulate type H vessel formation.

## Summary and outlook

With a continuous understanding of the pathogenesis of OP, we realize that bone metabolism and bone remodeling depend on the osteogenesis-angiogenesis coupling. Type H vessels are unique microvessels found within the bone, which play a crucial role in bone growth, repair, remodeling, and metabolism. So far, they have become a hot topic in studying the pathogenesis and treatment strategies of OP. The existing experimental studies on miRNA-regulated type H vessel formation explore the regulation mechanism of miRNAs in osteogenesis-angiogenesis coupling and provide potential targets for gene therapy for OP. Of course, the current experimental evidence is exciting, but most studies are still in their early stages, and we still need more basic research to explore some issues.

First, since Kusumbe and colleagues identified the type H vessels, scholars have characterized them based on their morphology, distribution, marker protein, surrounding cells, biological function, and the molecular mechanism of regulating type H vessel formation (71). However, our current knowledge of type H vessels remains limited. For example, the angiogenesis of H-type vessels and their functional relevance in bones at different age periods (infant, childhood, young and middle-aged, and old age) need further observation and comparison, especially the relationship among E-type, H-type, and L-type vessels. Type H vessels facilitate differentiation into arteries than type L vessels to participate in the local vascular network formation in bone tissue, which contributes to the formation of new bone by promoting the invasion of osteoprogenitors into the bone defect area (18). Type H endothelial cells play an essential function in the angiogenesis and differentiation of type H vessels. In addition, they can secrete many factors that stimulate the proliferation and differentiation of osteoprogenitors to regulate osteogenesis. Therefore, type H endothelial cells play a key role in connecting the processes of osteogenesis-angiogenesis coupling. Some scholars have reported various methods for isolating type H endothelial cells. For instance, Yang and colleagues used fluorescence-activated cell sorting (FACS) to sort the type H endothelial cells from bone marrow cells (34). Kusumbe and colleagues isolated these cells from bone tissue using Magnet-Activated Cell Sorting (MACS) after staining with the Endomucin antibody (17). Recently, He and colleagues used this method to harvest and culture type H endothelial cells *in vitro* to evaluate the cellular behavior and angiogenesis process (He et al., 2023). However, we haven’t been able to systematically observe and assess their cellular behavior, angiogenesis process, and the secretion mechanism of growth factors through experiments of the cultured endothelial cells *in vitro*. Scholars can use immunostaining to observe the abundance of the type H vessels or the expression of specific proteins of interest in OP rodent models or patients with OP (42). However, there are currently no reports on the testing method of surveying the changes in the morphology of subcellular organelles, inflammatory factors levels, and expressions of miRNAs, apoptosis-related genes, autophagy-related genes, and ferroptosis-related genes in the endothelial cells in the immunostained vessels. Hence, we also need more feasible experimental techniques to explore the reasons for the reduction of type H vessels in bones during the development of OP.

Second, miRNAs are involved in the cell signal transduction during osteogenesis-angiogenesis coupling, including HIF-1α/VEGF, Notch, and Wnt/β-catenin signaling pathways (72). In particular, the crosstalk between HIF-1α and Notch signaling is essential for type H vessel formation. In addition to the miRNAs introduced in this mini-review, other miRNAs may also regulate type H vessel formation, such as miRNA-210, miRNA-21, miRNA-199, etc. Moreover, the type H endothelial cells can produce and release exosomes containing miRNAs, but we still lack information about the functions of these exosomal miRNAs in type H vessel formation. Here, we concluded that miRNAs have two opposite effects on H-type vessel formation: promoting or inhibiting effect (**Table 1**). Therefore, we are required to choose the most suitable miRNAs as the breakthrough point in OP treatment. In addition, We need to consider the targeted and effective regulation of miRNAs *in vivo* and the development of miRNA carriers. Previous studies have shown that exosomes can serve as miRNA carriers and even as a cell-free treatment strategy for OP. However, the sorting mechanism for exosomal miRNAs is complicated, and the sorting process may be affected by intracellular and extracellular environments, which will lead to harvested exosomal miRNAs regulating many different signaling pathways to generate unexpected effects on recipient cells. Moreover, the research and development of engineered exosomes have always faced challenges, such as heterogeneity of exosomes, donor cell sources, isolation and purification methods, storage conditions, and drug loading efficiency (69).

**Table 1:** Literature examples of miRNAs in type H vessel formation.

mRNA	Type of mode	Affected molecules	Mode of action	Role in type H vessel formation	References
miRNA-7b	Mice, Endothelial progenitor cells	PDGF-BB	Reduce DC-STAMP to inhibit the formation of mature osteoclasts	Facilitation	32
miRNA-49~195	Mice, Bone marrow endothelial cells	HIF-1α	Inhibit P4HTM to maintain HIF-1α protein activity	Facilitation	34
miRNA-136–3p	Mice	PTEN	Bind to the 3′-UTR of PTEN mRNA	Facilitation	58
miRNA-143	Mice, Bone marrow endothelial cells	HDAC7	Bind to the 3′-UTR of HDAC7 mRNA	Facilitation	53
miRNA-149–5p	Mice, Mouse endothelial cell line C166	VEGF	Target Rap1a and Rap1b to inhibit VEGFR2 phosphorylation	Inhibition	39
miRNA-188–3p	Mice, Human microvascular endothelial Cell line-1	ITGβ3	Bind to the 3’UTR of ITGβ3 mRNA	Inhibition	46
miRNA-214–3p	Mice, Human umbilical vein endothelial cells	VEGF	Target RNA binding protein Quaking to reduce the expression and secretion of VEGF	Inhibition	38
miRNA-375	Mice	CTGF	Regulate miR375/CTGF signaling	Inhibition	42

Last but not least, both rodents and humans have type H vessels, and previous experimental studies on rodents provided some evidence that specific miRNAs can positively impact treating OP by regulating type H vessel formation. Still, the skeletal structures, bone remodeling processes, and genetics of humans and rodents differ (29). Therefore, we need to conduct more clinical experiments to study the role of miRNA-regulated type H vessel formation in OP patients. In addition, we need to evaluate the possible side effects of type H vessel formation in treating OP. For example, type H vessel formation may induce vascularization of subchondral bone, leading to osteoarthritis, or disturb the spatial arrangement of H-type blood vessels, leading to bone loss (72).

In short, our review shows that miRNAs play a crucial role in the osteogenesis-angiogenesis coupling, and miRNA-regulated type H vessel formation is involved in the pathogenesis of OP, which could become a breakthrough point in the therapy of OP. These findings will give us some valuable insights for future studies. Before translating the current achievements created in animal models into clinical practice, miRNA-based gene treatment strategies for OP still faced many challenges, and we should encourage further research efforts in this direction to find feasible solutions.

## Author contributions

DZ: Writing – original draft. YW: Writing – original draft. ZZ: Writing – original draft. LW: Writing – original draft. CL: Writing – original draft. YJ: Writing – review & editing, Writing – original draft.
